# “Property Phase Diagrams” for Compound Semiconductors through Data Mining

**DOI:** 10.3390/ma6010279

**Published:** 2013-01-21

**Authors:** Srikant Srinivasan, Krishna Rajan

**Affiliations:** Combinatorial Sciences and Materials Informatics Collaboratory, Department of Materials Science and Engineering, Iowa State University, Ames, IA 50011, USA; E-Mail: srikants@iastate.edu

**Keywords:** III–V materials, semiconductor compounds, bandgap engineering, crystal stoichiometry, structure-property relationships, phase diagrams, high dimensional data, data mining, materials informatics

## Abstract

This paper highlights the capability of materials informatics to recreate “property phase diagrams” from an elemental level using electronic and crystal structure properties. A judicious selection of existing data mining techniques, such as Principal Component Analysis, Partial Least Squares Regression, and Correlated Function Expansion, are linked synergistically to predict bandgap and lattice parameters for different stoichiometries of Ga*_x_*In_1−*x*_As*_y_*Sb_1−*y*_, starting from fundamental elemental descriptors. In particular, five such elemental descriptors, extracted from within a database of highly correlated descriptors, are shown to collectively capture the widely studied “bowing” of energy bandgaps seen in compound semiconductors. This is the first such demonstration, to our knowledge, of establishing relationship between discrete elemental descriptors and bandgap bowing, whose underpinning lies in the fundamentals of solid solution thermodyanamics.

## 1. Introduction

Design and characterization of materials has traditionally been approached using thermodynamic principles of free energy to capture the relationships between various thermodynamic properties through phase diagrams [1]. Such descriptions are obtained from *continuum representations* of bulk materials [2] and are often adequately expressed in terms of low order polynomial equations involving phenomenological parameters obtained heuristically or as fit to experiments [3]. However, it is widely recognized that such an approach tends to become approximate with the rapid discovery of new and complex materials, especially in the nanoscale regime. A classic example is the “effective-mass” description of semiconductor materials that starts losing relevance with the loss of periodicity at the nanoscale level, compounded with additional effects such as defects, doping, strain, *etc.* A natural solution to address the challenges of characterizing such complex materials across the misfit scale is to shift towards an atomistic description such as using first principles techniques [4]. However, despite rapid advances in computing, the first principles-based techniques for predicting properties of materials is extremely time consuming. Also, in many cases, the search process for new materials itself requires some direction. The problem becomes quite acute when dealing with multicomponent alloys that are potential candidates for many interesting applications. Thus, there is a lack of systematic guidelines that can allow experimentalists to investigate interesting composition spaces. Consequently the experimental approach has been to utilize a high throughput sample creation from different elements as a means of screening materials.

Here, we implement a different strategy [5] for materials modeling, wherein we seek to establish structure property relationships, *i.e.*, behavioral relationships between known *discrete scalar descriptors* associated with crystal and electronic structure, and the observed properties of the material. From this we can extract design rules that allow us to quantitatively describe the exact role of specific combination of materials descriptors towards governing a given property, such as the bandgap. This information could then be linked to a targeted first principles modeling step to provide a physical interpretation of mechanisms controlling bandgap.

To drive home this point we select techniques from existing work on different data-mining approaches and demonstrate in the Ga*_x_*In_1−*x*_As*_y_*Sb_1−*y*_ system that an initial set of 21 elemental descriptors can be reduced to a set of five critical descriptors that capture the widely studied “bowing” [6] of energy bandgaps in compound semiconductors. Our primary focus in this paper is to demonstrate that using a judicious combination of materials informatics techniques can provide a novel bottom-up viewpoint of property phase diagrams for complex materials.

## 2. A High Dimensional Data Approach to Bandgap Engineering

### 2.1. Negotiating through Continuum Representations—e.g., Correlated Function Expansion

The conceptual and mathematical development of correlated function expansion (CFE) has already been in use for some time now [7]. We summarize the technique briefly and review how such a technique can be applied to investigate properties throughout the composition space of complex materials. The underlying principle of CFE is that, when dealing with complex physical and chemical systems with dependencies on multiple independent and correlated components, the effects of these components on a particular property, e.g., bandgap, can be deduced from a “systematic procedure to render a high dimensional composition space down to a rapidly convergent hierarchical sequence of lower dimensional subspaces” [7]. A rigorous description of each of these subspaces can then be combined to estimate the material property value anywhere in the entire composition space.

Following the work in [8], we consider the example of the quaternary semiconductor alloy Ga*_x_*In_1−*x*_As*_y_*Sb_1−*y*_. The material property of interest (in this case the bandgap or lattice constant) is expressed as *ξ*(*x*) where *x* = {*x*_1_, *x*_2_,…, *x_N_*} is the collection of *N* component fractions. In the CFE, the model output property for a multicomponent system *ξ*(*x*) = *ξ*(*x*_1_, *x*_2_,…, *x_N_*) is expressed as a hierarchical correlated function expansion in terms of the input composition variables,

$$\xi (x)=\xi_{0}+\overset{N}{\underset{i=1}{\sum }}\xi_{i}(x_{i})+\overset{N}{\underset{1\leq i<j\leq 1}{\sum }}\xi_{ij}(x_{i},x_{j})+\cdots +\xi_{1,2,3,\ldots ,N}(x_{1},x_{2},\ldots x_{N})$$

Here, *ξ*_0_ is a constant, *ξ_i_*(*x_i_*) describes the independent role of the *i^th^* component, *ξ_ij_*(*x_i_*, *x_j_*) gives the correlated action of the variables *x_i_* and *x_j_*, *etc.* In the case of Ga*_x_*In_1−*x*_As*_y_*Sb_1−*y*_ this quaternary compound can be chemically resolved into constituent binary and ternary combinations. The constant *ξ*_0_ would relate to the constituent binary compounds (Table 1) while the function *ξ_i_*(*x_i_*) would relate to the next higher order term, *i.e.*, the constituent ternary compounds.

**Figure 1:**
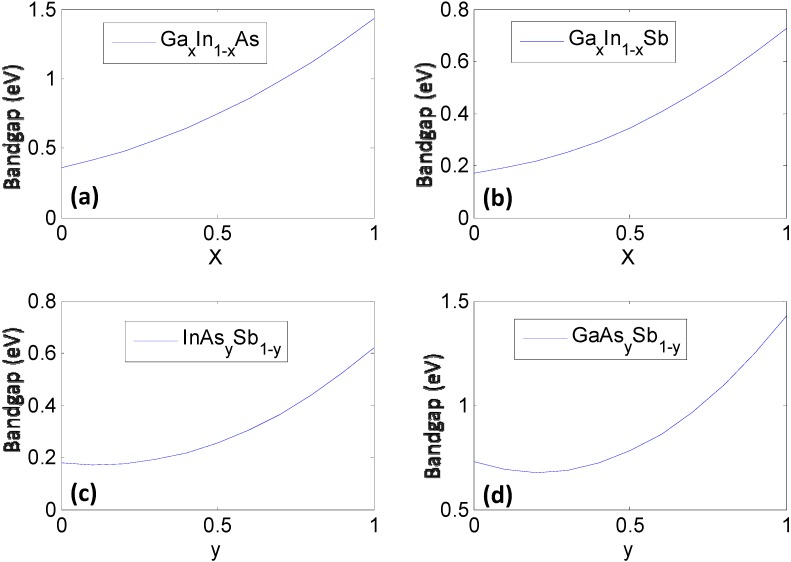
The constituent ternary compounds that combine in different ratios to form the quaternary semiconductor Ga*_x_*In_1−*x*_As*_y_*Sb_1−*y*_. Here, the bandgap of each ternary compound is plotted as a function of stoichiometry using phenomenological expressions (Table 2) obtained from fits to experiments (**a**) Ga*_x_*In_1-*x*_As; (**b**) Ga_x_In_1-x_Sb; (**c**) InAs*_y_*Sb_1-*y*_; and (**d**) GaAs*_y_*Sb_1-*y*_.

Although this equation looks similar to the standard Taylor series expansion, the functional form of the correlation terms can be highly nonlinear making it different. A truncation of the CFE, even to first order, can be nonlinear due to the nonlinear nature of the functions *ξ_i_*(*x_i_*) (Figure 1) as can be seen from the functional expressions in Table 2. In the context of bandgap it is this non-linear nature that is widely referred to as “bowing”, *i.e.*, the bandgap of an alloy does not change linearly as a function of the fraction of its constituent elements. The deviation from Vegard law behavior that is associated with the bowing is manifested through a complex combination of microstructural phenomena such as phase separation, clustering and spinodal decomposition. Subsequent sections of this study show how, through data mining, we can identify key parameters associated with the electronic structure of elements that contribute to the bowing behavior.

**Table 1:** Bandgap and lattice constant of binaries (*ξ*_0_).

Binary	Bandgap (eV)	Lattice Constant (Å)
GaAs	1.43	5.653
InAs	0.36	6.058
GaSb	0.68	6.095
InSb	0.17	6.478

**Table 2:** Bandgap and lattice constant of ternary compounds [9] (used in constructing *ξ_i_*(*x_i_*)).

Ternary	Bandgap (eV)	Lattice Constant (Å)
Ga*_x_*In_1−*x*_As	0.61*x*^2^ + 0.46*x* + 0.36	6.058 − 0.405*x*
Ga*_x_*In_1−*x*_Sb	0.415*x*^2^ + 0.139*x* + 0.172	6.478 − 0.383*x*
InAs*_y_*Sb_1−*y*_	0.58*y*^2^ − 0.14*y* + 0.18	6.478 − 0.420*y*
GaAs*_y_*Sb_1−*y*_	1.2*y*^2^ − 0.5*y* + 0.73	6.095 − 0.442*y*

**Figure 2:**
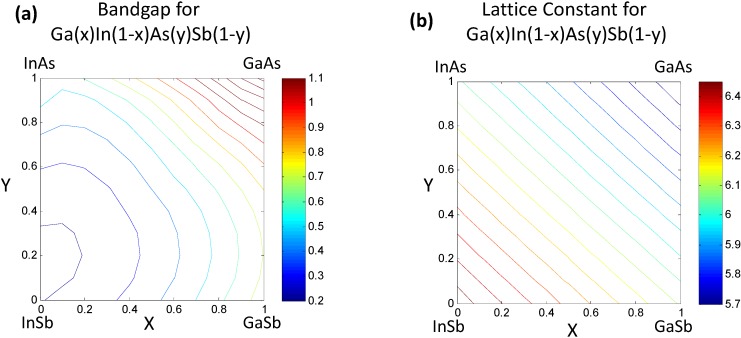
(**a**) Estimated bandgap for the quaternary semiconductor alloy Ga*_x_*In_1__−*x*_As*_y_*Sb_1__−*y*_ following the correlated function expansion (CFE) procedure in [8]. The contours truncated at 1.1 eV represent iso-“bandgap” regions; (**b**) Estimated lattice constants for the Ga*_x_*In_1−*x*_As*_y_*Sb_1−*y*_.

Once the values for the constant *ξ*_0_ and non-linear functional forms of *ξ_i_*(*x_i_*) are obtained, the bandgap/lattice constant *ξ*(*x*) for the quaternary combination can be determined. The details of the work are presented in [7,8]. We provide a simple reproduction of the results in this paper. For the reader’s convenience we would like to mention that the mathematics of the CFE formulation, in this case, essentially leads to calculation of the bandgap of the quaternary semiconductor Ga*_x_*In_1−*x*_As*_y_*Sb_1−*y*_ as an interpolation of the values obtained from the ternary compound equations (Table 2) with the constant binary compound values as the boundary conditions. The result shown in Figure 2a represents the estimated bandgap throughout the composition space of the quaternary compound. The contour lines represent regions having the same bandgap. The corners represent the values of the binary compounds, which form the “boundary condition” for the system, while the line joining any two binary compounds along the edges represent the bandgap for a ternary compound and visually follows the trend plotted in Figure 1. The bowing seen in Figure 2 obviously arises from the basis functions plotted in Figure 1, which are obtained as phenomenological fits to experiment and inherently have bowing incorporated in them. In the case of the lattice constants in Figure 2b, it can be seen that the relationships are very linear because they are based on a Vegard’s Law treatment. In the next section we will present treatment of this problem at a lower level of abstraction, namely using a set of elemental descriptors that form a discrete set, to determine the cause of the bowing.

### 2.2. Data Mining on Discrete Data

When dealing with a discrete data approach for exploring the property space of complex materials like Ga*_x_*In_1−*x*_As*_y_*Sb_1−*y*_, the strategy is to first identify a set of descriptors or parameters associated with the fundamental elements (in this case Ga, As, In, Sb).

These descriptors need not be related themselves except for the fact that they each describe some physical characteristic that may be relevant to our desired property (e.g., bandgap). The question of which and how many descriptors to choose is a topic that has been extensively studied in [10,11,12,13,14,15]. Here we follow the procedure adopted in [11]. The properties analyzed (listed in Table 3) were collected primarily from [16,17]. The primary challenge when considering a variety of descriptors of the elements is the significant multi-dimensionality. A variety of relationships can exist between descriptors, many of which may not be evident. The challenge then is to develop a representation of the elements, which captures the complex and multiple relationships.

**Table 3:** High dimensional representation of constituent atomic elements allowing the linking of various electronic properties with structural/crystal properties (a total of 21 descriptors in this case).

Element	MB	AN	MP	PR	*N*v	RH	CR	PEN	SH	HV	AW
Ga	1.7	31	302.93	1.695	3	−6.3	1.25	1.81	0.37	258.7	69.723
In	1.63	49	429.32	2.05	3	−2.4	1.5	1.78	0.23	231.5	114.818
Sb	2.14	51	903.89	1.765	5	−198	1.41	2.05	0.21	77.14	121.757
As	2.27	33	1090	1.415	5	450	1.21	2.18	0.33	34.76	74.92159

MB = Martynov-Batsanov electronegativity [(eV)^1/2^]; AN = Atomic Number; MP = Melting Point (K); PR = Pseudopotential core radii sum; *N*_V_ = Valence electron number; RH = Hall Coefficient (10^-11^ m^3^C^-1^); CR = Covalent Radius (Å); PEN = Pauling electronegativity; SH = Specific Heat (J/gK); HV = Heat of Vaporization (kJ/mol); AW = Atomic Weight.

**Table 3:** *Cont*.

Element	C	Sig	DT	FIP	SIP	EU	WF	AR	BP	D
Ga	25.86	0.0678	320	6	26.51	20.51	4.2	1.22	2676	5.907
In	26.74	0.116	108	5.78	24.64	18.86	4.12	1.63	2353	7.31
Sb	25.23	0.0288	211	8.64	25.1	16.46	4.55	1.82	1908	6.691
As	24.64	0.0345	282	9.81	30	20.19	5.2	1.25	889	5.78

C = Heat Capacity (J/mol-K); Sig = Electrical Conductivity (10^6^/cm-ohm); DT = Debye Temp (K); FIP = First Ionization Potential (eV); SIP = Second Ionization Potential (eV); EU = Effective U (eV); WF = Work Function (eV); AR = Atomic Radius (Å); BP = Boiling Point (K); D = Density at 293 K (g/cm³).

#### 2.2.1. Dimensionality Reduction of Discrete Data—e.g., Principal Component Analysis

**Figure 3:**
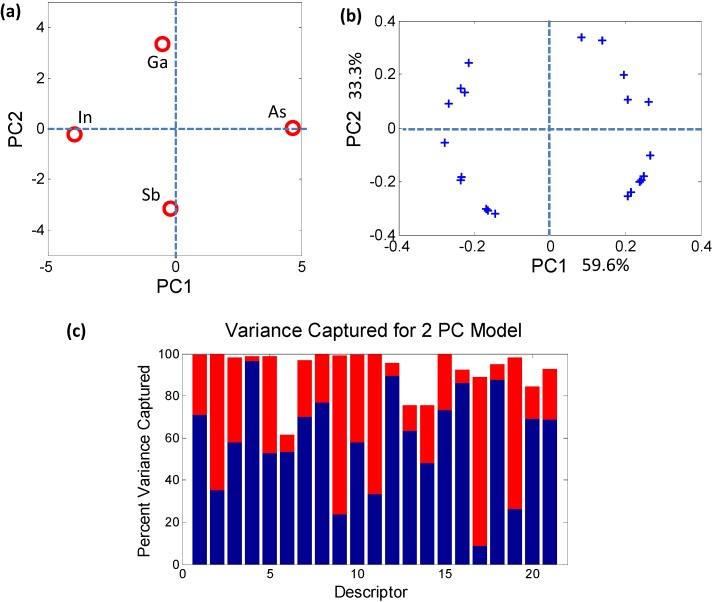
(**a**) Principal component analysis (PCA) *scores plot* demonstrating contrasting behavior of the individual elements that combine to form the quaternary semiconductor Ga*_x_*In_1−*x*_As*_y_*Sb_1−*y*_; (**b**) The circular arrangement of PCA *loadings plot* shows that each of the 21 descriptors plays a role in distinguishing between the atomic elements; (**c**) The histogram captures the contribution of each elemental descriptor towards each PC. The total variance captured by the first 2 PCs (~93%) is sufficient to describe the sample space.

The descriptor reduction method used here is the principal component analysis (PCA) [18,19,20]. PCA provides a projection of complex datasets onto a reduced, easily visualized space (Figure 3) while ensuring a minimization of loss of information. By capturing the correlated behavior of the descriptors PCA allows transformation of the original high dimensional coordinate system onto a reduced set of axes called *principal components* (PCs). Each newly constructed axis (or PC) is orthogonal to every other PC, thus capturing unique information.

**Figure 4:**
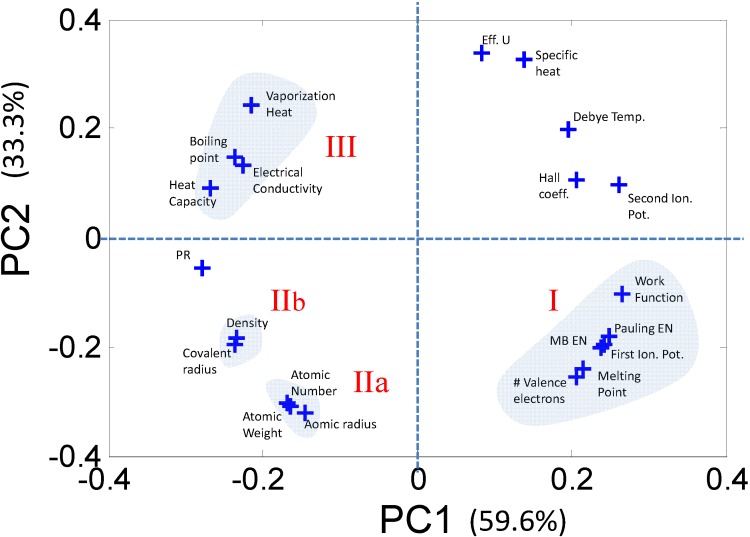
A close inspection of the loadings plot reveals that the 21 descriptors can be grouped into clusters comprising correlated variables. Each such cluster can be represented by a single descriptor, thus greatly reducing the dimensionality of the problem.

The PCs do not necessarily have an obvious physical meaning, but rather are a combination of descriptors which explain the largest variation in the data. In mathematical terms, PCA decomposes the original data matrix containing the elements (usually termed as samples) and the associated properties of the elements (usually termed as descriptors) into individual scores and loadings matrices. The scores values classify the samples in the PC space (Figure 3a) in terms of their dependence on the descriptors, *i.e.*, they effectively estimate the effect of one particular combination of descriptors on the samples. Similarly, the loadings values classify the descriptors (Figure 3b) in the PC space in terms of their separation of the elements. The advantage of PCA is that, since each PC uniquely captures the effect of a certain combination of relevant descriptors, typically a few PCs are sufficient for describing a system. For example, in the bivariate histogram in Figure 3c where the blue regions correspond to PC1 and the red regions correspond to PC2, the two PCs together capture ~93% of the variance of the data in Table 3. Therefore, a dataset of *n*-dimensions (21 initial descriptors in this case) can be reduced to a few dimensions (2 PCs) while capturing ~93% of the original information. The reduction in dimensionality makes trends and correlations, which are “hidden” in the data, become easily visualized and described in PC space as can be seen in Figure 4.

Once the correlations in the data are captured, each correlated group can be represented by a single descriptor that can be investigated closely to determine if it contributes to a structure-property relationship. Similarly, the descriptors which are diagonally opposite in the PC space are negatively correlated and can also be reduced into a single descriptor. Following the procedure in [11] we use a reduced set of five descriptors: (1) Martynov-Batsanov electronegativity (EN); (2) Atomic Number (AN); (3) Melting Point (MP); (4) Zunger’s Pseudopotential radii (PR); and (5) Number of Valence electrons (*N*_V_).

#### 2.2.2. Characterizing Ternary Compounds Using the Reduced Set of Elemental Descriptors

We now show how the discrete data description at the elemental level can be combined to encompass complex materials. We would like to reiterate here that the overall goal is to link the elemental descriptors of Figure 4 to the “bowing” of bandgaps in bulk semiconductors (Figure 2). To do so, we first derive a new set of discrete values for the ternary compounds in Figure 2, using the same descriptors as was used for their constituent elements. The parameterization of these descriptors for the ternary compounds is done using a relatively simple strategy originally proposed by Villars *et al.*, which involves a linear weighting model [21]. The formulations are given below for ternary compounds of type *A_x_* *B_y_* *C_z_* if *x* ≤ *y* ≤ *z* and *x* + *y* + *z* = 1:

- *EN* = 2*x*(*EN_A_* − *EN_B_*) + 2*x*(*EN_A_* − *EN_C_*) + 2*y*(*EN_B_* − *EN_C_*)
- *AN* = *x*(*AN*)_*A*_ + *y*(*AN*)_*B*_ + *z*(*AN*)_*C*_
- *MP* = *x*(*MP*)_*A*_ + *y*(*MP*)_*B*_ + *z*(*MP*)_*C*_
- *PR* = 2*x*(*PR_A_* − *PR_B_*) + 2*x*(*PR_A_* − *PR_C_*) + 2*y*(*PR_B_* − *PR_C_*)
- *N_v_* = *x*(*N_v_*)_*A*_ + *y*(*N_v_*)_*B*_ + *z*(*N_v_*)_*C*_

**Figure 5:**
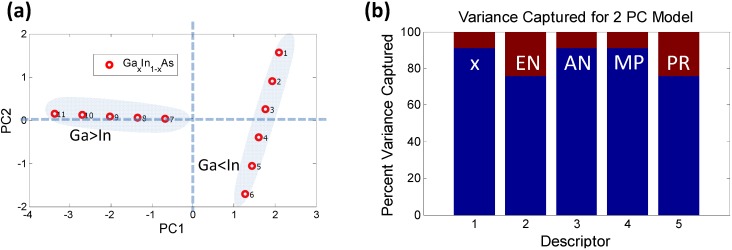
PCA analysis using the reduced dimensionality of descriptors (**a**) Scores plot for Ga*_x_*In_1−*x*_As: The data-points labeled 1 through 11 represent increasing concentration of Ga (*i.e.*, *x* = 0, 0.1, …, 1). It is clear that the samples where the In concentration exceeds the Ga concentration form a separate “orthogonal set”; (**b**) The possible reasons for such behavior are captured by the histogram indicating that descriptors 2 (EN) and 5 (PR) potentially play a role since they contribute significantly to PC2 (which is orthogonal to PC1).

In order to determine the effect of these descriptors on the properties of a ternary compound, say e.g., Ga*_x_*In_1−*x*_As, we generate a dataset of properties for different stoichiometries of the compound (for *x* = [0,1] in steps of 0.1) using the rules mentioned above. It is seen that the quantity *N_v_* remains a constant, independent of *x*. Therefore, it plays no role and can be dropped. A PCA analysis of the remaining descriptors combined with the stoichiometry parameter “*x*” is shown in Figure 5.

**Figure 6:**
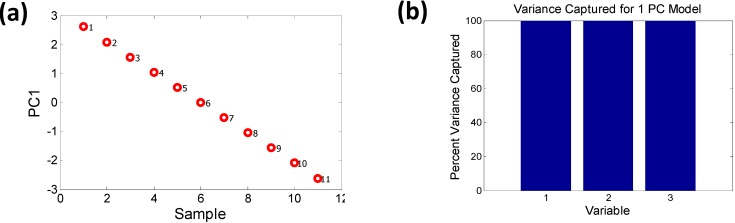
The removal of EN (Martynov-Batsanov electronegativity) and pseudopotential radius (PR) from the set of descriptors **(a)** removes the orthogonality and **(b)** the remaining descriptors are captured by a 1 PC model (*i.e.*, PC1 = 100% variance).

A two PC model captures nearly 100% of the variance. The scores plot shows how the samples can be grouped into two sets, one with majority Ga concentration and the other with majority In concentration, forming two distinct “phases”. One of the phases depends strongly on PC1 while the other varies with PC2. There is a possibility that such “phase” formation might contribute to bowing of the bandgap. The variance plot shows that the likely causes might be descriptors 2 (EN) and 5 (PR), since they contribute more significantly to PC2. Descriptors 1, 3 and 4 show an almost similar trend, as expected, since AN and MP vary linearly with stoichiometry. If we remove the descriptors 2 and 5 from the initial data set and run a PCA solely on descriptors 1, 3 and 4 it is seen that these descriptors follow the same pattern and are captured by just PC1 with a 100% variance, as shown in Figure 6.

### 2.3. Relating the Elemental Descriptors to Bandgap Bowing

We now discuss how one can relate the effect of the discrete elemental descriptors, discussed in the earlier section, to the continuum representation for bandgap given by the expressions in Table 2. The technique we adopt is Partial Least Squares (PLS) regression [22,23] using the elemental descriptors as “predictor variables” and the bandgap as a “predicted variable”. The working of PLS is quite similar to PCA, whereby the dataset is reduced into a set of orthogonal vectors that eliminate the effect of latency and collinearity. In order to predict the behavior of an output quantity (predicted variable) as a function of input variables (predictor quantities) an initial “training” data set is created that finds a relationship between the predictor and predicted variables by maximizing the covariance between them.

**Figure 7:**
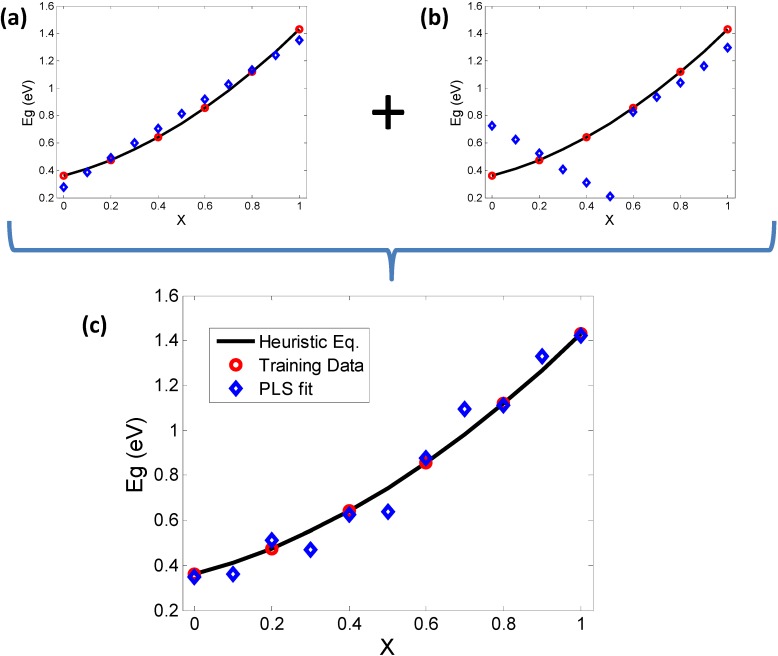
PLS (NIPALS) validation of PCA results and investigation of correspondence between bowing and elemental descriptors. The heuristic equations are plotted as a continuum (black line) and some samples (red circles) are chosen to train the Partial Least Squares (PLS) model. The prediction of PLS (blue diamonds) are then plotted for comparison. (**a**) Using the descriptors governed by PC1 (X, AN, MP) alone results in an inverse bowing trend; while (**b**) using MBEN and PR alone (PC2) shows orthogonal trends in the PLS prediction, clearly not in line with the continuum trend; (**c**) A “complete” set of descriptors including the contributors to PC1 and PC2 shows how the combination of results of (**a**) and (**b**) add up to give a more realistic trend.

In order to generate such training data for Ga*_x_*In_1−*x*_As, we include an additional column representing the predicted quantity (bandgap), calculated from the expressions in Table 2 for the same range of compositions (*i.e.*, *x* = 0, 0.1, …, 1). In continuation with the PCA analysis in the earlier section we initially generate two PLS models (Figure 7a,b) one of which uses predictors X, AN and MP, while the other uses EN and PR. The predicted results are then compared with the nonlinear heuristic equation for bandgap of Ga*_x_*In_1−*x*_As. The first model shows a bowing trend in the opposite direction while the second one shows orthogonal behavior due to the effect of EN and PR. However, when all predictor variables are considered together, a more realistic trend begins to appear, showing that all the predictor variables indeed have some contribution to the bowing trend of bandgap. A similar analysis was carried out with the other combinations of ternary compounds, namely Ga*_x_*In_1−*x*_Sb, GaAs*_y_*Sb_1−*y*_ and InAs*_y_*Sb_1−*y*_, leading to identical results in all these cases.

It is important to note that each of the predictor variables is representative of a cluster of correlated variables as shown in Figure 4. The refinement of these descriptors and the potential discovery of new and yet to be anticipated descriptors can be accomplished through an ensemble of informatics based methods as we have shown in previous work on other classes of materials chemistries [24,25]. Such approaches will be explored in future studies. The next step is to determine the quantitative relation between each of these descriptors and the thermodynamics of the solid solubility problem, which we leave for future work. In summary, this study serves to emphasize the value of data mining methods for capturing the underlying physics of “bowing” of bandgaps, which can be generalized to capturing property phase relationships of complex materials starting from discrete elemental descriptors, thus providing a bridge for representations from discrete to the continuum.

## 3. Conclusions

This paper has demonstrated the potential of data mining to redefine how we view property phase relationships starting from a basic elemental description. The example of the quaternary semiconductor compound Ga*_x_*In_1−*x*_As*_y_*Sb_1−*y*_ was chosen to elucidate this point wherein, a combination of five elemental descriptors was shown to relate to the “bowing” of bandgaps of compound semiconductors. The mathematical techniques presented in this paper such as PCA, PLS and CFE are by no means exhaustive but rather are representative of a wider class of techniques that collectively form the field of materials informatics. Such a framework for establishing property phase relationships can be particularly relevant for the accelerated discovery of complex materials or to analyze complex nanostructured systems lacking periodicity due to a variety of effects. Further, from a basic science perspective it provides the opportunity to map the standard continuum representation of materials onto high dimensional discrete representation, thus providing the opportunity to investigate potentially unexplored structure-property relationships and novel underlying physics.
